# Influence of pH on Heat-Induced Changes in Skim Milk Containing Various Levels of Micellar Calcium Phosphate

**DOI:** 10.3390/molecules28196847

**Published:** 2023-09-28

**Authors:** Elaheh Ahmadi, Todor Vasiljevic, Thom Huppertz

**Affiliations:** 1 Advanced Food Systems Research Unit, Institute for Sustainable Industries and Liveable Cities, College of Health and Biomedicine, Victoria University, Melbourne, VIC 8001, Australia; elaheh.ahmadi@live.vu.edu.au (E.A.); todor.vasiljevic@vu.edu.au (T.V.); 2 FrieslandCampina, 3818 LE Amersfoort, The Netherlands; 3 Food Quality and Design Group, Wageningen University and Research, 6708 WG Wageningen, The Netherlands

**Keywords:** thermal stability, micellar calcium phosphate, adjustment, skim milk, FTIR

## Abstract

The present study investigated the effect of micellar calcium phosphate (MCP) content and pH of skim milk on heat-induced changes in skim milk. Four MCP-adjusted samples, ranging from 67 to 113% of the original MCP content, were heated (90 °C for 10 min) at different pH values (6.3, 6.6, 6.9, and 7.2), followed by determining changes in particle size, turbidity, protein distribution, and structure. The results demonstrate a strong effect of MCP level and pH on heat-induced changes in milk, with the MCP_67_ samples revealing the greatest thermal stability. Specifically, decreasing MCP content by 33% (MCP_67_) led to a smaller increase in non-sedimentable κ-casein and a lower decrease in αs_2_-casein concentrations after heating compared to other samples. Lower MCP content resulted in a moderate rise in the average particle size and turbidity, along with lower loading of β-turn structural component after heating at low pH (pH 6.3). Notably, MCP_113_ exhibited instability upon heating, with increased particle size, turbidity, and a significant decrease in non-sedimentable αs_2_-casein concentration, along with a slight increase in non-sedimentable κ-casein concentration. The FTIR results also revealed higher loading of intermolecular β-sheet, β-turn, and random coil structures, as well as lower loading of α-helix and β-sheet structures in MCP-enhanced skim milk samples. This suggests significant changes in the secondary structure of milk protein and greater formation of larger aggregates.

## 1. Introduction

Heat treatment is one of the most widely used unit operations in the dairy sector and includes different heat intensities, such as thermisation (~62–65 °C for 10–20 s), pasteurisation (~72–80 °C for 15–30 s), ultra-high temperature (UHT) treatment (~135–150 °C for 1–10 s), and sterilisation (~110–120 °C for 10–30 min) [1,2]. Heat treatment is used to eliminate pathogenic microorganisms, increase the shelf life of milk, and/or to impart some desirable functional properties during further industrial processing of dairy products [3]. During thermal processing, milk can be exposed to conditions which alter milk protein structure, solubility, and functionality. Whey proteins play a key role in the heat-induced destabilisation of skim milk, via interactions with the casein micelles and aggregation, but dissociation of individual caseins from the casein micelles are also similarly important. In addition to heating time and temperature, factors such as the pH of the milk and the concentration of the soluble calcium also influence thermal stability of milk proteins [4].

Milk salts play a significant role in heat-induced changes in milk, particularly in relation to the heat-induced interactions between denatured whey proteins and casein micelles [5]. Early investigations revealed that modifications in micellar calcium phosphate (MCP) levels within the casein micelle could enhance the thermal stability of skim milk [6]. These studies emphasized the importance of understanding the interplay between MCP levels and the thermal characteristics of dairy systems, offering insights into potential strategies for optimizing heat stability of milk [6,7]. For example, Fox and Hoynes [6] showed that heat coagulation time (HCT) of milk was extended upon reduction in the MCP content, while it was reduced upon enhancing the MCP content in comparison to the original skim milk. In addition, the HCT clearly showed a pH dependence, with the greatest stability moving slightly above the natural pH of milk. Singh and Fox [7] reported that MCP adjustment had a minor impact on the levels of non-sedimentable N but somewhat increased the concentration of non-sedimentable N-acetylneuraminic acid (NANA), which appeared to remain elevated upon heating. Anema and Li [8] also explored the impact of varying MCP levels and selected pH on heat-induced changes in reconstituted skim milk, finding that increased MCP levels had minimal impact on the casein micelle dissociation, whereas reduced MCP content resulted in greater micellar dissociation. At pH 6.5, they observed that levels of non-sedimentable k-CN were lower than those at pH 7.1 after heating at 90 °C for 10 min. Other caseins experienced the opposite behaviour—greater concentration at pH 6.5 than those at pH 7.1 [8]. These studies highlighted the importance of MCP in maintaining micellar structure and its impact on the stability of the micelle upon heating [6,7,8].

As has been stated previously [9], the heat stability of milk is influenced by various factors, including the structural integrity of the casein micelle, particularly the dissociation of κ-casein (k-CN), pH fluctuations that occur during heating [10], and the involvement of ionic calcium. However, so much has been left to be explored when it comes to these impacts, especially the combined effect of MCP adjustment and pH; thus, the investigation is still ongoing. In order to provide further understanding of the behaviour and conformational changes of skim milk during heating, we have designed our investigation to include varying levels of the MCP (67 to 113% of the original) using pH adjustment followed by dialysis and influence of subsequently varying pH levels, ranging from low (pH 6.3) to high (pH 7.2) [11]. This comprehensive approach would contribute to a more holistic understanding of the interplay between MCP content, pH, and heat-induced changes in skim milk, shedding light on its potential applications in the dairy industry.

## 2. Results

### 2.1. Calcium Content of MCP-Adjusted Skim Milk

The effect of MCP adjustment on total calcium in unheated skim milk samples is shown in Table 1. The total calcium content of skim milk in the control milk (MCP_100_) was ~30–31 mmol L^−1^ and adjustment of MCP content clearly influenced the amount of total calcium, as expected. The lowest calcium level was observed in the sample acidified to pH 6.1 prior to dialysis, i.e., sample MCP_67_, and the highest concentration of Ca was found in sample MCP_113_, which had been adjusted to pH 7.5 prior to dialysis. Adjustment of pH to 6.3, 6.6, 6.9, or 7.2 after dialysis had no major effect on the total calcium content (Table 1).

### 2.2. Physiochemical Changes in MCP-Adjusted Skim Milk Samples after Heat Treatment

**Figure 1:**
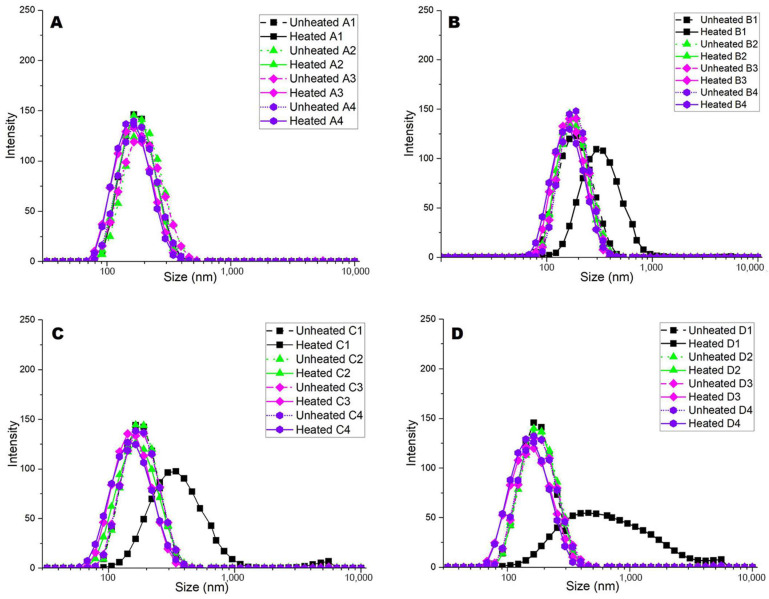
Particle size distribution of MCP-adjusted skim milk samples. MCP content was adjusted, varied by (**A**) 67% (MCP_67_), (**B**) 96% (MCP_96_), (**C**) 100% (MCP_100_ or Control), and (**D**) 113% (MCP_113_) relative to the control by either acidification or alkalisation followed by dialysis against original skim milk. Numbers 1 to 4 represent different readjusted pH, including 1 (pH 6.3), 2 (pH 6.6), 3 (pH 6.9), and 4 (pH 7.2). Graph is representative of two replicate samples.

The Z-average particle size of the samples is shown in Table 1, and particle size distributions of the samples are shown in Figure 1. The average particle size of sample MCP_100_ before heat treatment was ~163 nm (Table 1) and the particle size distribution of all dispersions had a main peak appearing between ~50 and ~400 nm, relating to that of native casein micelles in the MCP-adjusted skim milk samples (Figure 1). Indicatively from Table 1, turbidity was not affected; thus, the appearance of skim milk, such as its colour, did not change. The pH adjustment did not affect the average particle size and turbidity of unheated samples (Table 1).

After heating the control samples (MCP_100_) at pH 6.6, 6.9, or 7.2 at 90 °C for 10 min, particle size decreased by 12–22 nm (Table 1) and the particle size distribution shifted towards somewhat smaller particles (Figure 1). This heat-induced reduction in the average particle size of control milk is in agreement with the results previously reported [12]. Subjecting samples MCP_67_, MCP_96_, and MCP_113_ to heat treatment had no effect on the particle size at pH 6.6, whereas heating at pH 6.9 and 7.2 also resulted in a reduction in the particle size (Table 1). Heat treatment at pH 6.3 increased average particle size substantially, by 47–321 nm, with the extent of the heat-induced increase in particle size at this pH increasing with enhanced MCP content (Table 1; Figure 1). Along with these heat-induced increases in particle size, increases in turbidity were also observed (Table 1). The largest particle size (484 nm) and greatest turbidity (40.22) were observed for the milk sample MCP_113_ heated at pH 6.3 (Table 1). Although it is commonly observed that whey proteins undergo denaturation upon heating and interact with caseins [13], the substantial increase in particle size and turbidity observed after heat treatment of MCP_113_ at pH 6.3 exceeds the anticipated effects of whey protein aggregation alone in milk and rather suggests heat-induced aggregation of casein micelles.

### 2.3. Heat-Induced Changes in the Protein Distribution of MCP-Adjusted Skim Milk

**Figure 2:**
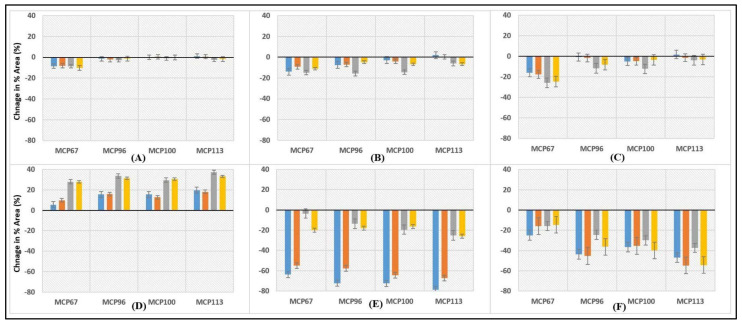
The total percentage areas of various proteins: (**A**) αs_1_-CN, (**B**) αs_2_-CN, (**C**) β-CN, (**D**) κ-CNs, (**E**) α-La, and (**F**) β-Lg in the supernatant of MCP-adjusted skim milk in relation to the entire milk were calculated by differentiating between heated and unheated skim milk samples. The MCP content of skim milk samples was adjusted, varied by 67% (MCP_67_), 96% (MCP_96_), 100% (MCP_100_ or Control), and 113% (MCP_113_) relative to the control by either acidification or alkalisation followed by dialysis against original skim milk. Supernatant obtained from unheated and heated milk at 90 °C for 10 min at pH 6.3 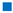; pH 6.6 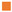; pH 6.9 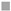; and pH 7.2 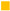.

The proportion of individual caseins and whey proteins in the supernatant of milk samples is shown in Table 2. Before heat treatment, ~4, 13, 18, 7, 88, and 72% of αs_1_-, αs_2_-, β-, and κ-CN, α-lactalbumin, and β-lactoglobulin, respectively, were found in the supernatant of sample MCP_100_ at pH 6.6. A reduction in MCP content resulted in an increase in the proportion of αs_1_-, αs_2_-, β-, and κ-CNs in the supernatant of unheated skim milk samples (Table 2), whereas non-sedimentable α-lactalbumin and β-lactoglobulin remained constant. MCP enrichment before heating did not significantly change the level of non-sedimentable αs_1_-CN at pH 6.6, whereas the levels of αs_2_-, β-, and κ-CNs in the supernatant decreased significantly compared to sample MCP_100_ (Table 2). Our results are in alignment with a previous study [7]. Adjusting pH to 6.3, 6.6, 6.9, or 7.2 of skim milk samples before heating only caused noticeable changes in levels of non-sedimentable αs_2_- and κ-CN. The level of non-sedimentable αs_2_-CN increased with decreasing pH (Table 2). In MCP-reduced unheated skim milk samples, non-sedimentable κ-CN increased when pH was reduced to 6.3, while its concentration was reduced in MCP-enhanced skim milk samples when pH was adjusted to 6.3. Adjusting pH above the natural pH of milk resulted in a reduction in non-sedimentable κ-CN independent of the MCP content (Table 2). Heat treatment of sample MCP_113_ at pH 6.3 and 6.6 resulted in a notable increase in non-sedimentable αs_1_-CN. A similar increase was noticed in MCP_100_ at pH 6.6. On the other hand, in all other skim milk samples, heat treatment decreased the levels of non-sedimentable αs_1_-CN (Table 2; Figure 2). Heat treatment also reduced levels of non-sedimentable α_s2_-CN and β-CN in all samples at all pH values, except for sample MCP_113_ at pH 6.3, in which the heat treatment led to an increase in the level of non-sedimentable α_s2_-CN (Table 2; Figure 2). In all heated samples, levels of non-sedimentable α_s2_-CN and β-CN appear to decrease with rise in pH, while non-sedimentable α_s1_-CN varied only slightly (Table 2), which is in agreement with the previously reported findings [8]. That study reported on the impact of heating temperature on properties of reconstituted skim milk with adjusted MPC content at pH 6.5 and 7.1 [8]. 

Contrary to the other caseins, the concentration of non-sedimentable κ-CN substantially increased after heat treatment, which was observed across all the samples (Table 2, Figure 2). Levels of non-sedimentable κ-CN in samples heated at pH 6.9 and 7.2 were considerably higher than those in the samples heated at pH 6.3 and 6.6. Only small differences at a given pH were observed between heated samples of different MCP contents (Table 2; Figure 2). These observations appear in agreement with a previous study, which showed a greater dissociation of κ-CN at pH 7.1 in comparison to that at pH 6.5 and governed by MCP content [8].

Levels of non-sedimentable α-La and β-Lg decreased after heat treatment, which is expected due to heat-induced denaturation and aggregation of whey proteins. MCP-adjustment had a notable impact on the distribution of the whey proteins compared to the control sample (MCP_100_) and levels of non-sedimentable whey proteins in heated samples decreased with increasing MCP content (Figure 2). In addition, it was observed that the final pH adjustment had a more pronounced impact on reducing the concentration of whey proteins at lower pH levels (pH 6.3), whereas, at higher pH levels (pH 6.9 and 7.2), the decrease in non-sedimentable whey protein was lower.

### 2.4. FTIR Fingerprinting

#### 2.4.1. Region I: Amide I (1700–1600 cm^−1^)

**Figure 3:**
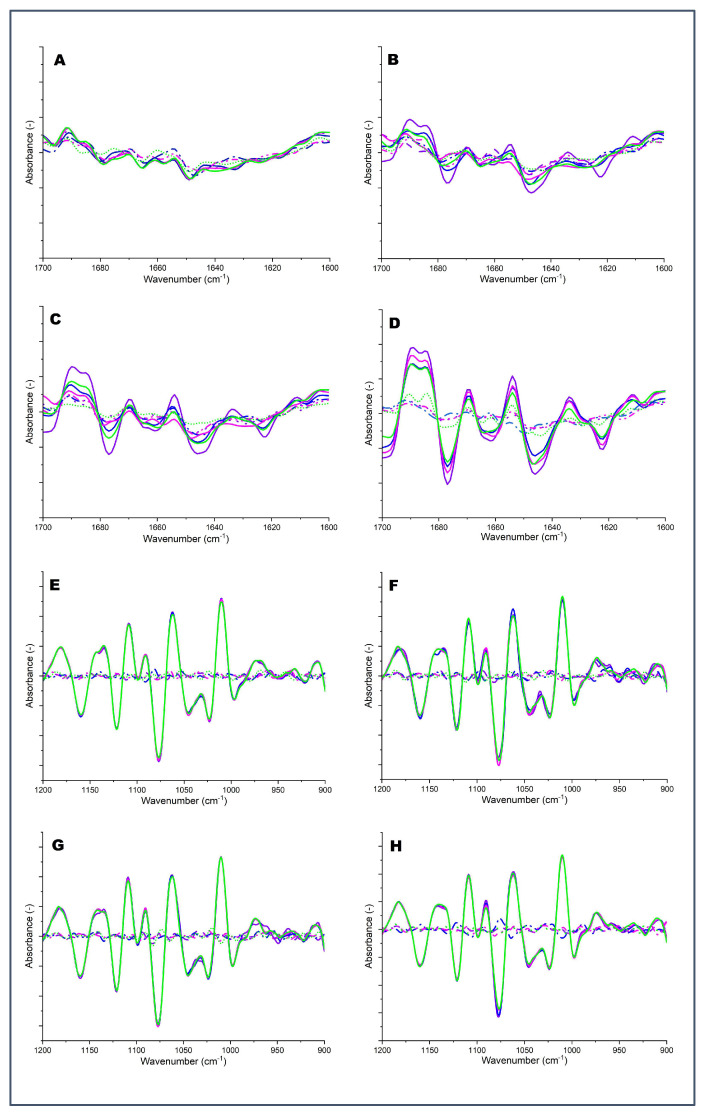
Second derivative of the FTIR spectra (Amide Ɪ region 1700–1600 cm^−1^) (**A**) MCP_67_, (**B**) MCP_96_, (**C**) MCP_100_, (**D**) MCP_113_ and the spectra region (1200–900 nm) (**E**) MCP_67_, (**F**) MCP_96_, (**G**) MCP_100_, and (**H**) MCP_113_. 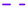 Unheated 1; 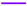 Heated 1; 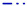 Unheated 2; 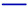 Heated 2; 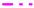 Unheated 3; 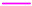 Heated 3; 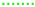 Unheated 4; 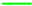 Heated 4. Numbers 1 to 4 represent 1: pH 6.3, 2: pH 6.6, 3: pH 6.9, and 4: pH 7.2.

**Figure 4:**
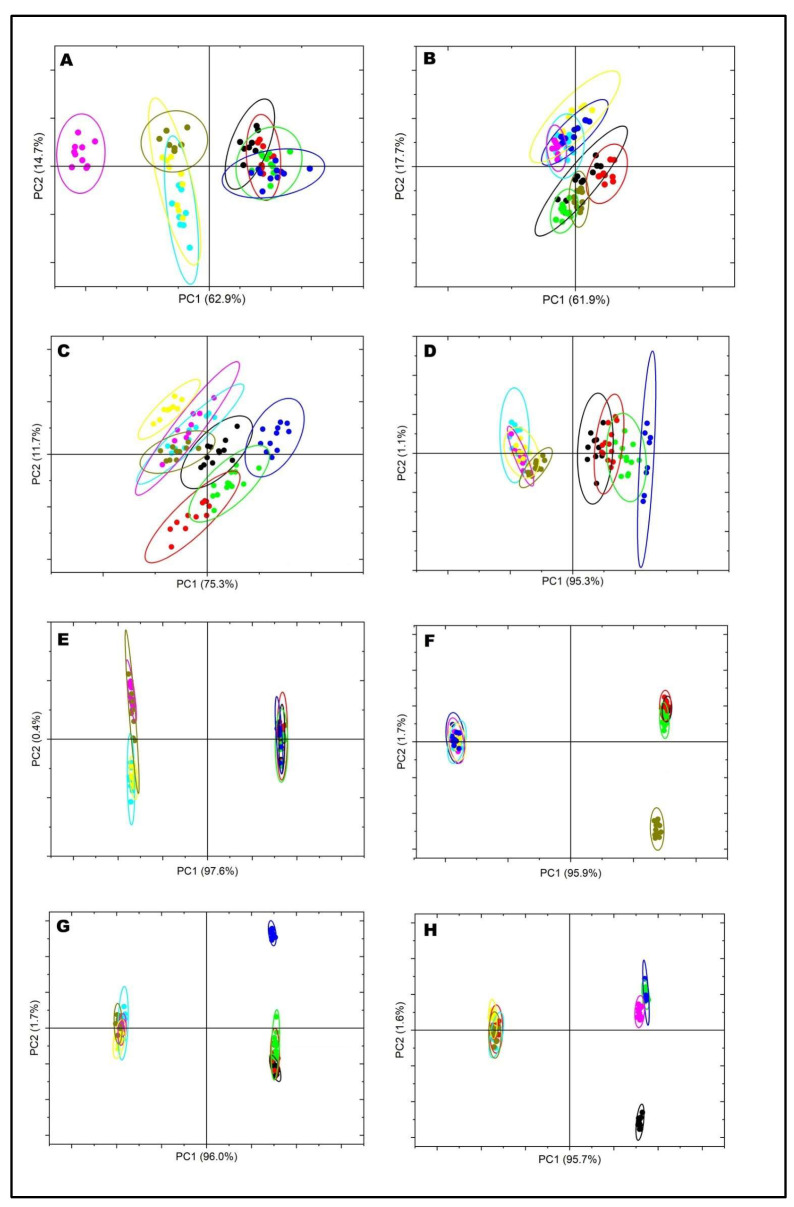
Principal component scores for Amide Ɪ region (1700–1600 cm^−1^) (**A**) MCP_67_, (**B**) MCP_96_, (**C**) MCP_100_, (**D**) MCP_113_ and the spectra region (1200–900 nm) (**E**) MCP_67_, (**F**) MCP_96_, (**G**) MCP_100_, and (**H**) MCP_113_. 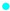 Unheated 1; 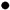 Heated 1; 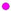 Unheated 2; 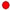 Heated 2; 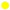 Unheated 3; 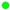 Heated 3; 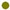 Unheated 4; 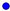 Heated 4. Numbers 1 to 4 represent 1: pH 6.3, 2: pH 6.6, 3: pH 6.9, and 4: pH 7.2.

FTIR was used to fingerprint the changes in the secondary structure of milk proteins as a result of the heat treatment (Figure 3). The FTIR spectra in the region between 1700 and 1600 cm^−1^ arising from the absorption associated with the amide I region (1700 and 1600 cm^−1^) has six features: intermolecular/aggregated β-sheets (1700–1681 cm^−1^), β-turns (1680–1660 cm^−1^), α-helix (1652–1641 cm^−1^), random coils (1640–1631 cm^−1^), intramolecular β-sheet (1630–1620 cm^−1^), and side chains (1618–1608 cm^−1^) [14]. Figure 3 shows the dominant effect of heat treatment on the secondary structure in skim milk samples, particularly in the area of intermolecular β-sheets (1700–1681 cm^−1^), presumably due to heat-induced aggregate formation. PCA analysis confirmed the distinction between heated and unheated samples by effectively separating them (Figure 4). Heated MCP-enhanced skim milk samples exhibited greater loading for intramolecular β-sheet structures, whereas, in the MCP-depleted samples, the changes were not substantial compared to unheated original skim milk samples (Figure 3). Furthermore, the intramolecular β-sheet structures also displayed pH dependence; at pH 6.3, there was a noticeable shift in loading with increased peak intensity, indicating a higher presence of intermolecular β-sheet structures (Figure 3).

Heat-induced changes in milk caused an increase in the intensity of β-turn peaks (1660–1680 cm^−1^), as observed in FTIR analysis. The presence of β-turns in milk proteins is considered a consequence of protein unfolding from higher-order structures [15]. Adjustment of MCP content influenced the intensity of peaks in the β-turn region. In heated MCP_113_ samples, an increase in β-turn was observed (Figure 3). However, in the heated MCP-depleted skim milk samples (MCP_67_), there were no significant changes compared to unheated milk samples. The changes in β-turns were also influenced by the pH adjustment. The intensity of the β-turn peak showed a negative correlation with pH, indicating that, at lower pH levels (pH 6.3), there was a higher presence of β-turns (Figure 3). In addition, there was a substantial decrease in the intensity of the peak in the α-helix region (1652–1641 cm^−1^) in heated MCP-enhanced samples (MCP_113_), whereas the random coils (1640–1631 cm^−1^) exhibited a remarkable increase after heating (Figure 3). Notably, the absorbance at the random coil area was significantly lower in skim milk samples with a reduced MCP content, which corresponded to the higher heat stability of these samples. The most prominent change in the α-helix and random coil was observed when the sample pH was adjusted to 6.3 (Figure 3). Moreover, decreases in ordered secondary structure, mainly β-sheet (1630–1620 cm^−1^), upon heating were expected. Interestingly, the intensity of the peak in highly MCP-reduced samples (MCP_67_) was close to the original unheated milk, while β-sheet rose in the control and MCP-enriched samples, which has been related to substantial aggregation [15]. In particular, the lowest loading of intramolecular β-sheet (1630–1620 cm^−1^) was at lower pH (6.3) in heated skim milk samples, which exhibited substantial denaturation and aggregation of proteins. The rise in the loadings around 1618–1608 cm^−1^ has also been attributed to disruption of intramolecular hydrogen bonds within a secondary structure [16], leading to formation of new stronger intermolecular hydrogen bonds (Figure 3).

#### 2.4.2. Region II (1200–900 cm^−1^)

Region II (1200–900 cm^−^^1^) of analysis was able to detect changes in both milk carbohydrates and minerals, as shown in Figure 4. The intensity of peaks around 1200 and 1100 cm^−^^1^ depicts various carbohydrate vibrations, with lactose being the primary component [17], which did not change substantially as a result of MCP adjustment, pH adjustment, or heat treatment. Decreasing the pH caused a slight decrease in the loading at 1089–1058 cm^-1^, which indicates the dissociation of phosphate from casein micelle. Conversely, higher pH demonstrated increased absorption (Figure 3). The absorption values of this peak (1089–1058 cm^−^^1^) were found to be lower for heated milk when compared to unheated milk (Figure 3). PCA also confirmed the difference in this region by classifying the samples into two groups. Principal Component 1 (PC1) separated the heated skim milk samples from unheated samples (Figure 4).

Changes in the intensity of the peaks around 995 and 987 cm^−^^1^ in Figure 3 depict changes in stretching vibrations of the –PO3^2−^ moiety of phosphoserine [17] and are related to the dissociation of MCP. As expected, MCP-reduced skim milk samples (MCP_67_) exhibited a lower intensity of peaks at 995–987 cm^−^^1^, while MCP-enhanced samples (MCP^113^) showed a higher intensity of peak around this area (995 and 987 cm^−^^1^). The pH adjustment to a low value (6.3) led to a decrease in the intensity of the peak (995 and 987 cm^−^^1^), which likely depicted the dissociation of MCP from the phosphoserine residues. Heat treatment also caused lower loading at 995 and 987 cm^−^^1^, which is indicative of the impact of heating on calcium solubility, as illustrated in Figure 3. The PCA analysis also supported the disparity in the mineral regions by dividing the samples into two distinct groups. PC1 was able to differentiate between heated skim milk samples and unheated samples (Figure 4).

## 3. Discussion

In addition to time and temperature, the thermal stability of milk is strongly influenced by pH and minerals [2]. The present study examined the thermal behaviour of the MCP-adjusted skim milk samples, with the MCP content varying from 67 to 113% of that found in the control milk at pH adjusted to 6.3, 6.6, 6.9, or 7.2. Based on the previous research, pH adjustment followed by dialysis is a recognized method for modulating micellar calcium content of milk [11]. Our preliminary study revealed that MCP-reduced skim milk samples appeared to retain casein micelle integrity when MCP content was reduced by up to 33% (MCP_67_). Such an adjustment of the MCP content within the casein micelle has been reported to affect the thermal stability of skim milk expressed as HCT [6]. The relationship was inverse—lowering or enhancing MCP content prolonged or shortened HCT, respectively [6]. Singh and Fox [7] further emphasized the inverse relationship between levels of micellar calcium phosphate (MCP) and the thermal stability of skim milk at alkaline pH. They also stated that the behaviour of k-CN may not be fully driven by MCP content and suggested that some of this protein may be attached to the micelle through some other electrostatic attractions [7]. Anema and Li [8] investigated how changing MCP levels and pH affected heat stability of reconstituted skim milk. They observed that lowering of MCP levels led to substantial micellar dissociation, while MCP levels greater than the original MCP content had very little effect on the micelle integrity [8]. When considering the impact of heating on skim milk, it is important to note that the reduction in calcium phosphate solubility occurs as the temperature increases [5,18]. κ-CN can interact with whey proteins on the surface of casein micelles [19] and in the serum phase of milk in the form of soluble complexes [20]. In the present study, κ-CN displayed more pronounced changes compared to the other caseins upon MCP adjustment at different pH levels (Figure 2). The concentration of non-sedimentable κ-CN significantly increased by reducing the MCP content, similar to results previously reported [8]; at the same time, no significant change in particle size and turbidity was observed at pH above 6.6; however, both parameters increased significantly when the samples were heated at low pH (Table 1). These observations indicate that the MCP appears to be an important factor in maintaining the micellar integrity, while some other factors are involved when it comes to pH-dependant behaviour. However, it seems obvious that milk proteins behave differently below the natural pH of milk that could also be indirectly governed by the MCP content.

It is well known that during heating of milk at 90 °C, two main phenomena take place—k-CN dissociates from the micelle, which is illustrated by its concentration rise in the serum phase (Table 2) and denaturation of whey proteins, which consequently create sedimentable and non-sedimentable aggregates with k-CN and/or themselves (Table 2). The presence of aggregated proteins is evidenced by the level of loading at 1700–1681 cm^−1^, suggesting the formation of intramolecular β-sheet aggregates (Figure 3). The lower loading at these wavelengths appears to be related to the reduced MCP content.

Furthermore, lowering of the MCP content leads to greater micellar dissociation and greater content of individual caseins in the soluble phase, which may also start participating directly or indirectly in these complex reactions (Table 2). For example, the concentration of non-sedimentable αs_2_-CN decreased after heating at pH 6.9 and 7.1. While its concentration also decreased in comparison to the original levels, the levels of non-sedimentable αs_2_-CN remained high after heating at pH 6.6 and 6.3 (Table 2, Figure 2). This suggests that sedimentable aggregates created at elevated pH contained more αs_2_-CN linked via its sulfhydryl groups to other particles, including either complexes with k-CN and whey proteins or reattachment to the casein micelle. The particle size (and turbidity) remained fairly consistent across most of the MCP/pH range, which changed once pH before heating was adjusted to 6.3. A notable trend was observed that the particle size and turbidity were MCP-dependent at this pH—being larger at higher MCP content (Table 1). Reduction in all heat-reactive proteins, including αs_2_-CN, k-CN, β-lactoglobulin, and even α-lactalbumin, in the soluble phase after heating is indicative of excessive aggregation and precipitation after centrifugation. A threefold increase in the particle size after heating of MCP_113_ at pH 6.3 is suggestive of casein–casein interactions via covalent or calcium-induced bridging due to higher Ca content in this sample (Table 1). Calcium forms complexes with the phosphate groups found on αs_2_-CN [21] and the binding of Ca to αs-CNs is stronger than binding to other caseins [22], which could address the decline in non-sedimentable portion of this protein in this sample compared to the MCP_67_ sample heated at the same pH. The FTIR analysis also illustrated a notable decrease in the intensity of the peak in the α-helix region (1652–1641 cm^−^^1^), accompanied by a remarkable increase in the presence of random coils (1640–1631 cm^−^^1^) after increasing the MCP content in skim milk followed by heating (Figure 3). It can be related to substantial unfolding of the secondary structure of the proteins, leading to denaturation of whey proteins or greater contribution of caseins, which are mainly characterised by a large proportion of random structures [23].

Interactions among milk proteins at low MCP level heated at pH 6.3 appear to be different. While the increase in particle size was expected due to minimisation of the surface charge, the rise was not as great as the one observed with the enhanced MCP sample. The fundamental difference is in the greater presence of non-sedimentable caseins resulting from reduced MCP content that may have governed aggregation behaviour of whey proteins. For example, less β-lactoglobulin and α-lactalbumin was incorporated into sedimentable aggregates when the sample with reduced MCP content was heated at pH 6.3 (Table 2). It is known that β- and αs_1_-CNs possess a high chaperone-like activity [24]. This activity is not a true chaperon activity, as it only governs the aggregation step of the reaction, leading to the formation of smaller non-sedimentable particles (Table 2, Figure 2) [25]. The decrease observed in β-turns (1660–1680 cm^−^^1^) upon reducing the MCP content and heating (Figure 3) may be thus attributed to reduced interactions between κ-casein and β-lactoglobulin (predominantly). These interactions are known to contribute to the formation of loops, triple helices, and turns [23].

Previous studies indicated that the heat stability of milk was substantially reduced at lower pH levels [6,7]. As shown in our study, initial pH adjustment causes substantial structural modifications of proteins, which likely heightens their reactivity during heat treatment. In addition, heating also leads to a slight drop in the pH of the milk due to the release of hydrogen ions [10]. Therefore, it was expected that lowering the pH would intensify a greater presence of κ-CNs in the serum before heating, along with enhanced dissociation of casein micelles during heating at this pH, leading to considerable aggregation of casein micelles. Previous studies have also reported an increased amount of whey proteins complexed with the micelles at lower pH [19,26]. However, this general trend was not really observed in the samples with lower MCP content heated at low pH, as the absence of larger particles indicated different aggregation patterns, resulting in smaller particles. If the standard test for measuring heat stability of milk was used, in this case, measurement of HCT, that would likely indicate greater stability due to the absence of visible aggregation.

## 4. Materials and Methods

### 4.1. Sample Preparation

**Figure 5:**
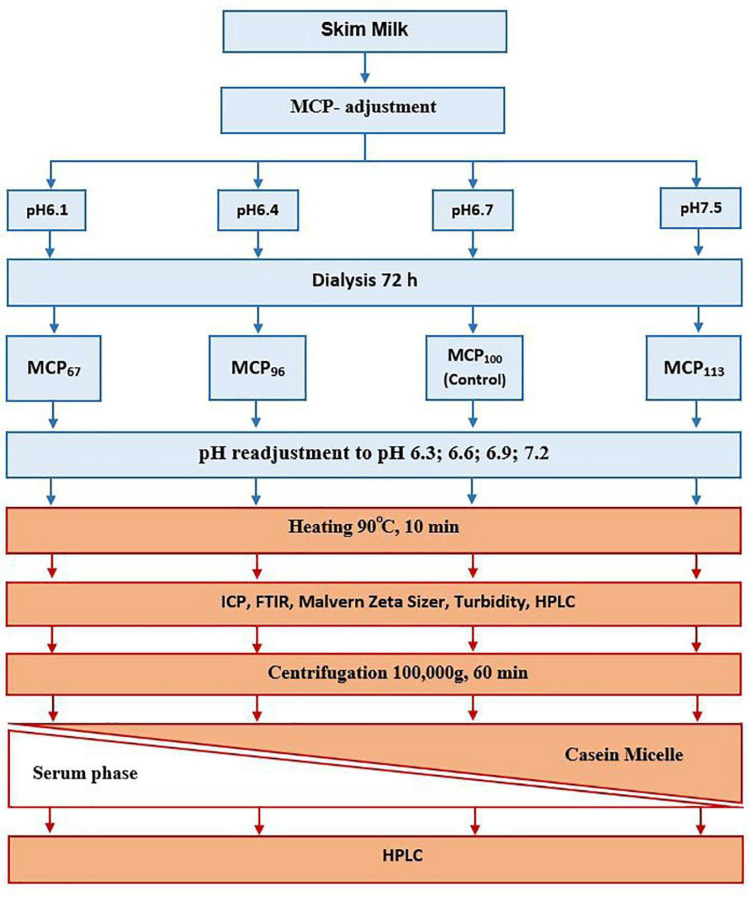
Experimental design of the study.

The design of the experimental work is schematically presented in Figure 5. Freshly pasteurised skim milk was obtained from a commercial dairy (Warrnambool Cheese and Butter—Saputo, Warrnambool, Australia). To prevent bacterial growth, sodium azide (0.02%, *w*/*w*) was added to the milk. According to a protocol described previously [11], a predetermined amount of glucono delta-lactone (GDL) or 1.0 M NaOH was added to lower or increase the pH of skim milk to 6.1, 6.4, 6.7, or 7.5. After pH was stabilised, the samples were dialysed using a high retention seamless cellulose dialysis tubing (14 kDa MWCO, Sigma-Aldrich, St. Louis, MO, USA) against 2 × 20 volumes of original pasteurised skim milk for 72 h at 5 °C [11,27]. After dialysis, the samples were removed from the dialysis tubing. The sample coding was based on the estimate of micellar Ca relative to that of the control as described previously [27]. Following this procedure, the MCP_67_ sample thus had its MCP content reduced from the initial 100% to 67%. Conversely, in the MCP_113_ sample, the MCP content increased by 13% compared to the initial MCP amount of 100%. Then, the pH of the samples was adjusted to 6.3, 6.6, 6.9, or 7.2. Once the pH was stable, the samples were heated in an oil bath set at 90 °C. The time to reach 90 °C was ~3 min, after which samples were held for a further 10 min at this temperature and then cooled to 20 °C by immersion in an ice bath.

### 4.2. Sample Fractionation

Unheated and heated milk samples were fractionated by ultracentrifugation at 100,000× *g* for 1 h at 20 °C in a Beckman Ultra L-70 centrifuge (Beckman Coulter, Australia Pty. Ltd., Gladesville, Australia). After the ultracentrifugation, the clear supernatant was carefully collected from each tube using a syringe.

### 4.3. Sample Analysis

#### 4.3.1. Calcium Content

The total calcium (Ca) of the unheated MCP-adjusted pH-adjusted skim milk samples was determined using an inductively coupled plasma atomic emission spectrometer (ICP-AES, ICPE-9000 system, Shimadzu Corporation, Kyoto, Japan), following the method of Bijl et al. [2].

#### 4.3.2. Turbidity

Turbidity of the samples was measured at 860 nm using a 1 mm pathlength quartz cuvette using a UV-Visible spectrophotometer (Biochrom Ltd., Cambridge, UK).

#### 4.3.3. Particle Size Distribution

Particle size analysis of samples was performed by dynamic light scattering (Zetasizer-Nano, Malvern instruments Ltd., Malvern, UK) at a scattering angle of 90° and temperature was maintained at 25 °C. Samples were diluted in simulated milk ultrafiltrate (SMUF) [28] in a ratio of 1:100 [2].

#### 4.3.4. High-Performance Liquid Chromatography (HPLC)

Individual caseins in whole samples and ultracentrifugal supernatants were analysed by reversed-phase high-performance liquid chromatography (RP-HPLC) at room temperature using a Shimadzu HPLC system (Model Prominence-i, LC-2030 C, Shimadzu Corporation, Kyoto, Japan) with a Varian 9012 system controller (Agilent Technologies Inc., Santa Clara, CA, USA) coupled with an RI detector (Varian, Palo Alto, CA, USA, 9050) and a C4 column (Aeris WIDEPORE, 150 mm × 4.6 mm, 3.6 μm particle size, 300 Å porosity, Phenomenex, Torrance, CA, USA) using pretreatment and elution conditions as described previously by Aprianita et al. [29]

#### 4.3.5. Fourier Transform Infrared Spectroscopy (FTIR)

FTIR measurements were conducted using an FTIR spectrometer (PerkinElmer, Boston, MA, USA) in the range of 4000–600 cm^−1^. At the start of measurement, the background spectrum was scanned with a blank (SMUF) and corresponding ultracentrifugal supernatant using the same instrumental conditions as for the sample spectra acquisition [17]. FTIR experiments for each sample were replicated twice (on two sets of samples). Principal component analysis (PCA) was employed to better understand the changes in the conformation of caseins induced by different environments. FTIR data processed as described previously was reported with the Origin software (Origin Pro 2021, v. 95E, OriginLab Corporation, Northampton, MA, USA) [17].

### 4.4. Statistical Analysis

All experiments assessed the impact of heat treatment on the selected parameters in the MCP-adjusted skim milk. SPSS software (v. 26, IBM Inc. Chicago, IL, USA) was used to conduct a two-way analysis of variance (ANOVA) to establish differences among means, followed by Tukey’s multi-comparison of the means. The level of significance was set at *p* < 0.05. The design was replicated three times on three different occasions.

## 5. Conclusions

The result of this study shows a strong influence of MCP adjustment on the thermal behaviour of milk proteins and, consequently, on the heat stability of milk. The key finding achieved by this study was that the sample containing the lowest MCP content appeared to have a high level of intact casein micelles and likely the greatest thermal stability among all samples. The pH level of the milk was also observed to have a direct relationship with its heat stability, with lowered pH levels resulting in reduced heat stability. MCP adjustment leads to differing behaviour of individual caseins, which dissociate to a certain extent at lower MCP content, while remaining at high levels at an MPC level above the initial. This leads to different interactions among proteins in the soluble and colloidal phases and different outcomes. Smaller particle size is observed at low pH and low MCP content; thus, greater heat stability may be expected. Notably, this work provided for the first time an insight into the effect of heat treatment on the conformational changes of MCP-adjusted and enriched skim milk heated at different pH. This can assist in greater understanding of the functional properties of MCP-adjusted skim milk in industrial-scale dairy processing to achieve skim milk with improved thermal and structural stability.

## Figures and Tables

**Table 1:** Calcium concentration in milk before heat treatment, particle size of MCP-adjusted milk before and after heat treatment for pasteurised skim milk samples with their MCP adjusted from 67% (MCP_67_) to 113% (MCP_113_) by either acidification or alkalisation followed by exhaustive dialysis against bulk milk ^1,2^. For sample details, see Section 4.1 ^1^.

MCP-Adjusted Samples	pH	Total Ca (mmoL L^−1^)	Particle Size Unheated (nm)	Particle Size Heated (nm)	Turbidity Unheated (cm^−1^)	Turbidity Heated (cm^−1^)
MCP_67_	6.3	21.76 ± 0.05 ^E^	160 ± 1 ^Ab^	207 ± 1 ^Da^	0.29 ± 0.01 ^Ab^	0.53 ± 0.03 ^Ca^
	6.6	21.76 ± 0.11 ^E^	161 ± 2 ^Aa^	157 ± 1 ^Ea^	0.34 ± 0.01 ^Aa^	0.36 ± 0.02 ^Da^
	6.9	22.01 ± 0.05 ^E^	162 ± 1 ^Aa^	152 ±1 ^Eb^	0.27 ± 0.02 ^Ab^	0.32 ± 0.01 ^Da^
	7.2	21.81 ± 0.20 ^E^	160 ± 1 ^Aa^	150± 3 ^Eb^	0.26 ± 0.03 ^Aa^	0.29 ± 0.02 ^Da^
MCP_96_	6.3	25.70 ± 0.05 ^D^	161 ± 1 ^Ab^	296 ± 5 ^Ca^	0.29 ± 0.00 ^Ab^	0.90 ± 0.07 ^Ba^
	6.6	26.10 ± 0.00 ^CD^	161 ± 0 ^Aa^	156 ± 1 ^Ea^	0.32 ± 0.02 ^Aa^	0.35 ± 0.01 ^Db^
	6.9	25.90 ± 0.13 ^D^	161 ± 1 ^Aa^	154 ± 1 ^Eb^	0.31 ± 0.01 ^Aa^	0.32 ± 0.02 ^Db^
	7.2	24.53 ± 0.07 ^D^	162 ± 1 ^Aa^	149 ± 2 ^Eb^	0.26 ± 0.03 ^Aa^	0.33 ± 0.01 ^Db^
MCP_100_(Control)	6.3	27.85 ± 0.08 ^C^	162 ± 0 ^Ab^	325 ± 6 ^Ba^	0.36 ± 0.03 ^Ab^	0.94 ± 0.02 ^Ba^
	6.6	27.46 ± 0.05 ^C^	163 ± 1 ^Aa^	151 ± 1 ^Eb^	0.35 ± 0.04 ^Aa^	0.37 ± 0.02 ^Da^
	6.9	27.65 ± 0.05 ^C^	165 ± 1 ^Aa^	145 ± 1 ^Eb^	0.32 ± 0.02 ^Aa^	0.34 ± 0.01 ^Da^
	7.2	26.83 ± 0.55 ^C^	164± 1 ^Aa^	142 ± 2 ^Eb^	0.32 ± 0.02 ^Aa^	0.31 ± 0.00 ^Da^
MCP_113_	6.3	31.25 ± 0.18 ^A^	163 ± 1 ^Ab^	484 ± 3 ^Aa^	0.34 ± 0.02 ^Ab^	1.60 ± 0.04 ^Aa^
	6.6	32.31 ± 0.12 ^A^	165 ± 1 ^Aa^	160 ± 1 ^Ea^	0.35 ± 0.00 ^Ab^	0.42 ± 0.01 ^Da^
	6.9	30.46 ± 0.02 ^B^	165 ± 1 ^Aa^	147 ± 1 ^Eb^	0.34 ± 0.01 ^Aa^	0.38 ± 0.03 ^Da^
	7.2	32.76 ± 0.12 ^A^	165 ± 1 ^Aa^	143 ± 1 ^Eb^	0.34 ± 0.02 ^Aa^	0.33 ± 0.03 ^Da^

^1^ The subscripts indicate proportion of retained MCP relative to that of the control; ^2^ the capital letters indicate significant differences across entire rows. The small letters show significant differences between two correlated samples across the columns (*p* < 0.05).

**Table 2:** Influence of heat treatment (90 °C for 10 min) on levels of non-sedimentable caseins and whey proteins in MPC-adjusted skim milk adjusted to pH 6.3, 6.6., 6.9, or 7.2 prior to heating ^1,2^.

MCP-Adjusted Samples	pH	Milk Protein Concentration (%)											
		αs_1_-Casein		αs_2_-Casein		β-Casein		κ-Casein		α-Lactalbumin		β-Lactoglobulin	
		Unheated	Heated	Unheated	Heated	Unheated	Heated	Unheated	Heated	Unheated	Heated	Unheated	Heated
MCP_67_	6.3	14.2 ± 0.1 ^Ba^	5.7 ± 0.2 ^Ab^	27.5 ± 0.3 ^Aa^	13.6 ± 0.5 ^Ab^	36.9 ± 0.1 ^Aa^	20.8 ± 0.2 ^Ab^	21.4 ± 0.1 ^Ab^	26.9 ± 0.1 ^Ga^	84.0 ± 0.3 ^Ba^	20.2 ± 0.02 ^Gb^	69.4 ± 0.4 ^Da^	44.6 ± 0.2 ^Bb^
	6.6	14.0 ± 0.1 ^Bb^	5.8 ± 0.4 ^Ab^	20.3 ± 0.1 ^Ba^	11.1 ± 0.1 ^Bb^	36.3 ± 0.0 ^Aa^	18.6 ± 0.1 ^Bb^	15.1 ± 0.0 ^Cb^	24.9 ± 0.1 ^Ha^	79.1 ± 0.9 ^CDa^	24.2 ± 1.0 ^Fb^	70.0 ± 0.2 ^Da^	54.2 ± 0.3 ^Ab^
	6.9	14.5 ± 0.1 ^Ba^	5.8 ± 0.1 ^Ab^	16.1 ± 0.0 ^Ca^	1.3 ± 0.0 ^Db^	36.5 ± 0.1 ^Aa^	10.7 ± 0.1 ^Fb^	16.5 ± 0.1 ^Bb^	44.4 ± 0.1 ^Aa^	82.8 ± 0.2 ^Ba^	79.2 ± 0.4 ^Ab^	72.0 ± 0.2 ^Ca^	56.1 ± 0.8 ^Ab^
	7.2	16.1 ± 0.0 ^Aa^	5.8 ± 0.2 ^Ab^	13.1 ± 0.1 ^Da^	2.0 ± 0.1 ^Db^	36.3 ± 0.1 ^Aa^	11.6 ± 0.1 ^Fb^	15.8 ± 0.0 ^BCb^	43.7 ± 0.1 ^Ba^	80.4 ± 0.2 ^Ca^	60.4 ± 0.1 ^Eb^	70.8 ± 0.3 ^Da^	56.3 ± 0.1 ^Ab^
MCP_96_	6.3	5.2 ± 0.1 ^Ca^	3.8 ± 0.9 ^Bb^	16.1 ± 0.0 ^Ca^	8.6 ± 0.1 ^Cb^	17.4 ± 0.0 ^BCa^	16.8 ± 0.3 ^Cb^	7.6 ± 0.1 ^Db^	23.1 ± 0.1 ^Ja^	81.8 ± 0.1 ^Ca^	9.5 ± 0.1 ^Hb^	76.4 ± 0.1 ^BCa^	32.7 ± 0.3 ^Eb^
	6.6	5.9 ± 0.1 ^Ca^	3.7 ± 0.3 ^Bb^	16.3 ± 0.1 ^Ca^	9.2 ± 0.1 ^Cb^	18.5 ± 0.1 ^Ba^	16.8 ± 0.1 ^Cb^	7.3 ± 0.1 ^Db^	23.2 ± 0.0 ^Ja^	81.7 ± 0.1 ^Ca^	24.2 ± 0.2 ^Fb^	77.8 ± 0.1 ^Ba^	32.4 ± 0.1 ^Eb^
	6.9	5.1 ± 0.1 ^Ca^	2.2 ± 0.1 ^Cb^	17.0 ± 0.2 ^Ca^	1.3 ± 0.0 ^Db^	19.0 ± 0.1 ^Ba^	7.3 ± 0.1 ^Hb^	5.9 ± 0.0 ^DEb^	39.6 ± 0.1 ^Da^	83.7 ± 1.6 ^Ba^	70.1 ± 0.3 ^Bb^	69.2 ± 0.2 ^Da^	44.8 ± 0.4 ^Bb^
	7.2	5.5 ± 0.4 ^Ca^	4.2 ± 0.1 ^Bb^	6.7 ± 0.1 ^Ea^	1.9 ± 0.1 ^Db^	17.6 ± 0.2 ^BCa^	9.4 ± 0.1 ^Gb^	5.6 ± 0.1 ^DEb^	37.1 ± 0.1 ^Ea^	81.5 ± 0.1 ^Ca^	63.5 ± 0.5 ^Db^	67.8 ± 0.1 ^Ja^	31.5 ± 0.2 ^Eb^
MCP_100_(Control)	6.3	3.8 ± 0.1 ^Da^	3.7 ± 0.1 ^Ba^	11.1 ± 0.1 ^DEa^	8.3 ± 0.1 ^Cb^	18.0 ± 0.0 ^BCa^	12.8 ± 0.3 ^Eb^	4.2 ± 0.0 ^Eb^	19.7 ± 0.1 ^Ka^	83.0 ± 0.4 ^Ba^	10.5 ± 0.1 ^Hb^	76.3 ± 0.0 ^BCa^	39.8 ± 0.1 ^Cb^
	6.6	3.6 ± 0.1 ^Db^	4.0 ± 0.5 ^Ba^	12.9 ± 0.0 ^Da^	8.9 ± 0.0 ^Cb^	17.9 ± 0.1 ^BCa^	13.2 ± 0.1 ^Eb^	6.7 ± 0.1 ^Db^	19.4 ± 0.1 ^Ka^	87.6 ± 0.2 ^Aa^	23.1 ± 0.1 ^Fb^	72.2 ± 0.0 ^Ca^	37.0 ± 0.2 ^Db^
	6.9	3.5 ± 0.0 ^Da^	2.4 ± 0.3 ^Cb^	15.6 ± 0.1 ^Ca^	1.2 ± 0.0 ^Db^	19.8 ± 0.0 ^Ba^	7.5 ± 0.0 ^Hb^	6.3 ± 0.1 ^Db^	35.8 ± 0.0 ^Fa^	86.5 ± 0.1 ^Aa^	67.0 ± 1.4 ^Cb^	73.8 ± 0.1 ^Ca^	43.9 ± 0.2 ^Bb^
	7.2	3.9 ± 0.1 ^Da^	3.7 ± 0.1 ^Bb^	8.3 ± 0.0 ^Ea^	1.5 ± 0.0 ^Db^	12.1 ± 0.0 ^CDa^	8.5 ± 0.1 ^GHb^	5.3 ± 0.0 ^DEb^	35.8 ± 0.1 ^Fa^	79.9 ± 1.3 ^Ca^	63.4 ± 0.1 ^Db^	72.5 ± 0.3 ^Ca^	32.7 ± 0.1 ^Eb^
MCP_113_	6.3	3.0 ± 0.1 ^Db^	4.1 ± 0.1 ^Ba^	5.5 ± 0.2 ^EFb^	7.5 ± 0.1 ^Ca^	13.3 ± 0.1 ^Cb^	15.1 ± 0.1 ^Da^	4.2 ± 0.1 ^Eb^	23.9 ± 0.1 ^Ia^	88.8 ± 0.2 ^Aa^	10.2 ± 0.0 ^Hb^	83.9 ± 0.2 ^Aa^	37.1 ± 0.1 ^Db^
	6.6	3.4 ± 0.1 ^Db^	4.0 ± 0.1 ^Ba^	7.6 ± 0.1 ^Ea^	8.1 ± 0.1 ^Ca^	14.8 ± 0.1 ^Ca^	13.4 ± 0.1 ^Eb^	5.7 ± 0.1 ^DEb^	23.8 ± 0.1 ^Ia^	87.1 ± 0.9 ^Aa^	19.9 ± 0.2 ^Gb^	77.2 ± 0.2 ^BCa^	22.6 ± 0.1 ^Fb^
	6.9	3.6 ± 0.3 ^Da^	1.3 ± 0.1 ^Cb^	6.7 ± 0.1 ^Ea^	0.6 ± 0.0 ^DEb^	10.2 ± 0.1 ^Da^	6.3 ± 0.0 ^HIb^	3.5 ± 0.0 ^Eb^	40.8 ± 0.1 ^Ca^	88.7 ± 0.3 ^Aa^	63.6 ± 0.2 ^Db^	76.1 ± 0.1 ^BCa^	38.8 ± 0.0 ^Cb^
	7.2	3.0 ± 0.1 ^Da^	1.4 ± 0.1 ^Cb^	6.7 ± 0.0 ^Ea^	0.0 ± 0.0 ^Eb^	10.3 ± 0.1 ^Da^	7.3 ± 0.0 ^Hb^	3.8 ± 0.1 ^Eb^	37.0 ± 0.1 ^Ea^	86.1 ± 0.3 ^Aa^	60.3 ± 0.3 ^Eb^	78.8 ± 0.0 ^Ba^	24.6 ± 0.1 ^Fb^

^1^ The subscripts indicate proportion of retained MCP relative to that of the control; ^2^ the capital letters indicate significant differences across entire rows. The small letters show significant differences between two correlated samples across the columns (*p* < 0.05).

## Data Availability

Data are contained within the article.
